# Practical Notes on Diagnosis and Treatment

**Published:** 1913-02-15

**Authors:** 


### PRACTICAL NOTES ON DIAGNOSIS AND TREATMENT.

**Uræmia.**

In persistent uræmic convulsions 1 to 2 drachms of chloral hydrate may be given in the form of enema.

**Quiet Effusion into the Knee-Joints.**

This is seen both in girls at puberty and in women at the menopause. The treatment needed is attention to the general health and massage of the joints. The condition does not call for rest or for the use of splints.

**Chronic Polyarthritis.**

It is a great mistake to order a restricted diet for patients suffering from the so-called rheumatoid arthritis. Such patients, on the contrary, need a generous diet, containing a fair proportion of meat, and many are all the better for some good malt liquor.

**Food Fever.**

Recurring attacks of high temperature in children are sometimes due to excess of carbohydrate food; at least, they cease when starchy and saccharine materials are reduced to a minimum and the child is given broths, flesh, meat, etc., etc. An occasional dose of calomel is also a useful measure.

**Granular Kidney.**

Pleurisy, and even pericarditis, are sometimes the first apparent evidences of chronic renal disease; they may occur in such circumstances without fever. Their presence in a patient of middle age calls for careful examination of the urine, and almost without exception they carry a very bad prognosis.

**Treatment of Chorea.**

If arsenic is ordered in cases of chorea, it is well to precede it by a mild mercurial purge and by a stomachic mixture for, say, forty-eight hours. Then the arsenic may be pushed in rapidly increasing doses. An occasional attack of vomiting does not contra-indicate perseverance with the drug.

**Hyperpyrexia.**

For this serious complication of acute rheumatism the cold bath (65° F.) is demanded, the temperature being kept about 60° by the addition of ice. The patient should be immersed until his temperature has fallen 5° to 6°, unless there is shivering, when he should at once be removed. Failing the bath, an ice-pack or rubbing the surface with pieces of ice should be tried.

**Sciatica.**

Among methods of treatment sometimes successful in obstinate cases are the following: 1. Pilocarpine, $\frac{1}{3}$ grain every night injected subcutaneously in the painful area, and a minim of croton oil, followed by a saline purge once a week. 2. Colchicum, combined with free purgation; this method being most appropriate in middle-aged patients with high arterial tension. 3. Acupuncture, a long needle being passed into the nerve at about an inch above the level of the great trochanter and left *in situ* for twenty minutes.

**Hæmoptysis.**

Given the absence of mitral disease, hæmoptysis almost invariably ueans phthisis pulmonalis.

**Papain in Distended Stomach.**

Papain is often helpful in cases of sluggish digestion, with distention of the stomach; at the same time, a little water acidulated with hydrochloric acid may be sipped with and after the meal.

**Myopia.**

Myopic children should be prevented from an undue amount of near work, and the practice of distant vision should be encouraged, as by shooting at a target, flying kites, etc. Of course, such children need suitable glasses.

**Ichthyol at the Menopause.**

The flushings and other disturbances which often attend the menopause can be favourably influenced by ichthyol. This is especially true of eczema and other irritable conditions of the skin. The dose should be 2½, increased to 5 or even 10, grains after food thrice daily.

**Postural Albuminuria.**

This ought not to be treated by milk diet, restricted exercise, etc. The boy should lead an active, vigorous life, and should have a generous diet. If he is of the flabby anæmic type, iron, arsenic, or strychnine will do him good. Granted accuracy of diagnosis the prognosis is very good.

**High Arterial Tension.**

"A measure in which my experience has given me confidence for the reduction of high tension is the employment of mild mercurial aperients. The effect of purgatives on the tension is not proportionate to the amount of purgation, and the effect on the circulation of a single grain of some mercurial preparation, with slight aperient action, is much greater than repeated liquid motions induced by salines."—*Sir William Broadbent.*

**Rheumatic Fever.**

A good working rule is to order 20 grains sodium salicylate and 30 grains sodium bicarbonate every two hours until the pain is relieved. Then the dose may be given at four-hourly intervals until the temperature is normal; afterwards a reduced dose may be continued four times a day until all evidences of active rheumatism have disappeared. Salicin is by some preferred to the salicylate, and this specially applies to rheumatism in childhood.

**Saline Injections.**

A convenient place for introducing fluid below the skin is the front of the thigh, the puncture being made near the apex of Scarpa's triangle. This is much less disagreeable to the patient than the front of the chest or the axillary region. Instead of allowing the fluid to enter by gravity (a very slow process) it can be introduced through the needle by a clean Higginson's syringe. If the part distended by the fluid is firmly bandaged this latter is soon absorbed.

